# Cytokines in the Pathogenesis of Large Granular Lymphocytic Leukemia

**DOI:** 10.3389/fonc.2022.849917

**Published:** 2022-03-10

**Authors:** Colleen Isabelle, Amy Boles, Nitin Chakravarti, Pierluigi Porcu, Jonathan Brammer, Anjali Mishra

**Affiliations:** ^1^Division of Hematologic Malignancies and Hematopoietic Stem Cell Transplantation, Department of Medical Oncology, Sidney Kimmel Cancer Center, Thomas Jefferson University, Philadelphia, PA, United States; ^2^Division of Hematology, The Ohio State University, Columbus, OH, United States; ^3^Department of Cancer Biology, Sidney Kimmel Cancer Center, Philadelphia, PA, United States

**Keywords:** interleukins, growth factors, cytokines, LGLL, therapy

## Abstract

Large granular lymphocytic leukemia (LGLL) is a lymphoproliferative disorder of older adults characterized by the clonal expansion of cytotoxic T/natural killer cells due to constitutive pro-survival signaling. In recent years, it has become clear that cytokines and their receptors are aberrantly expressed in LGLL cells. The exact initiation process of LGLL is unknown, although several cytokine-driven mechanisms have emerged. Elevated levels of several cytokines, including interleukin-15 (IL-15) and platelet-derived growth factor (PDGF), have been described in LGLL patients. Evidence from humans and animal models has shown that cytokines may also contribute to the co-occurrence of a wide range of autoimmune diseases seen in patients with LGLL. The goal of this review is to provide a comprehensive analysis of the link between cytokines and pro-survival signaling in LGLL and to discuss the various strategies and research approaches that are being utilized to study this link. This review will also highlight the importance of cytokine-targeted therapeutics in the treatment of LGLL.

## Introduction

Large granular lymphocytic leukemia (LGLL) is a lymphoproliferative disorder of older adults characterized by the clonal expansion of effector cytotoxic T cells or natural killer (NK) cells. The WHO classifies LGLL into T-cell LGLL (~85% of all cases) and chronic NK-cell lymphoproliferative disorder (NK-CLPD also known as NK-LGLL) (~10% of all cases) (1). Although sometimes included in the LGLL family, aggressive NK-cell leukemia (ANKL) is a distinct neoplasm of NK cells that is nearly always associated with Epstein–Barr virus (EBV) infection and has a very poor prognosis (2). While T-LGLL and NK-LGLL are classified as separate disorders, their pathogenesis is essentially identical and therefore will be considered together in this review.

The exact cause of LGLL is unknown. To date, studies examining the biology of LGLL have identified several altered growth factors signaling pathways in these leukemic cells, which induce molecular aberrancies believed to play a role in the development of LGLL and in its clinical and laboratory manifestations. This review aims to provide an overview of the role of cytokines in the development of LGLL.

## Overview of Large Granular Lymphocytic Leukemia Development

The importance of cytokine dysregulation in LGLL pathogenesis has been well established (3). LGLL represents an expansion of activated cytotoxic lymphocytes that persist after antigenic stimulation. LGLL is initiated by an unknown pathogenic trigger or triggers to activate the initial immune cell reaction and increase the production of pro-inflammatory cytokines by LGL cells (4–7). This causes polyclonal reactive cell expansion. However, unlike the normal T-cell LGL expansions in response to antigen, which are controlled and resolved through T-cell apoptosis or differentiation into the memory T-cell pool, LGLL cells begin to clonally proliferate (6, 8). This dysregulated clonal expansion is currently attributed to alterations of multiple pro-survival and anti-apoptotic signaling pathways, especially constitutively active cytokine signaling (9). The major cytokine factors and their interactions with oncogenic signaling pathways in LGLL will be reviewed here.

## Aberrantly Expressed Cytokines in Large Granular Lymphocytic Leukemia

During disease development, LGLL cells may acquire the ability to sustain proliferative signaling by producing growth factors and their cognate receptors themselves, resulting in chronic autocrine proliferative stimulations (10, 11). LGLL cells can also respond to soluble growth factors present in the pro-inflammatory microenvironment (12). The cytokines that have emerged as major players in LGLL pathogenesis are presented below.

### Interleukin-15

Interleukin-15 (IL-15) is a 15-kDa, four-helix bundle cytokine that plays a crucial role in the development of innate immunity (13). It is central to NK cell and NK-T cell development and activation. IL-15 was discovered in 1994 as a T-cell proliferation factor that shared the interleukin-2 (IL-2) receptor βc and γ_C_ subunits (14). Signaling occurs through the IL-15Rαβγ heterotrimeric receptor complex that includes the shared βc and γ_C_ chains, as well as a private α receptor (15). The IL-15 gene consists of 9 exons spanning approximately 34 kb on chromosome 4q31 in humans and chromosome 8 in mice, with 73% conservation between species (13, 16). Both mice and humans have an alternatively spliced isoform of IL-15 that also encodes the mature IL-15 protein with potentially different secretion capacity (17). IL-15 has wide tissue distribution and is typically expressed by stromal cells, epithelial cells, and monocytes. However, it is not typically expressed by T cells. Expression of IL-15 by LGLL cells is abnormal and promotes LGLL cell survival (10). The role of IL-15 in the pathogenesis of LGLL has been well documented (3, 10, 18–20). IL-15 normally regulates T- and NK-cell activation, proliferation, and cytotoxicity. Zambello et al. (20) established that LGLL isolated from patients constitutively express all three of the IL-15 receptor components: IL-15Rα, βc, and ɣ_c_. The proliferation of LGLL cells constitutively expressing IL-15 receptors is enhanced by the addition of exogenous IL-15 *in vitro* and showed enhanced cytotoxic activity (20). LGLL cells have increased membrane-bound IL-15 on their surface as compared to healthy controls (21). Typically, IL-15 is presented in *trans*- to NK and T cells that express IL-2/15Rβɣ. It is therefore interesting that Chen et al. (18) demonstrated increased levels of soluble IL-15Rα (sIL-15Rα) in the serum of patients with LGLL as well as upregulated levels of IL-15Rα mRNA in patient peripheral blood mononuclear cells (PBMCs). They speculate that this increased sIL-15Rα in LGLL patient serum could be a product of increased enzymatic cleavage from cell surfaces or due to alternative splicing resulting in the soluble isoform. Chen et al. (18) also showed increased IFNɣ mRNA in PBMCs from T-LGLL patients, which is known to induce expression of IL-15Rα in monocytes. IL-15 signaling contributes to LGLL pathogenesis through several mechanisms including hypermethylating DNA, altering microRNA expression, and activating several oncogenic pathways such as Jak/STAT, Ras, PI3K, and NF-kB (10). Through these mechanisms, as further detailed in subsequent sections of this review, IL-15 promotes pro-survival and anti-apoptosis signaling in LGLL as a key player in the immunopathogenesis of this disease.

### Platelet-Derived Growth Factors

Platelet-derived growth factors (PDGFs) are produced by many different cell types, such as fibroblasts, endothelial cells, and macrophages. Overproduction of these factors is a known contributor to many types of cancer and disease (22, 23). The PDGFs are dimeric growth factors ranging in size from approximately 27 to 30 kDa. They activate two related transmembrane tyrosine kinase receptors, PDGF-α and PDGF-β, leading to downstream effects (22, 23). The five PDGF isoforms are PDGF-AA, PDGF-BB, PDGF-AB, PDGF-CC, and PDGF-DD. All ligands except PDGF-DD activate PDGF-α receptor dimerization in the cell. Similarly, all ligands except the PDGF-AA can activate the α and β receptors in cells (22).

Network modeling of LGLL survival pathways by Zhang et al. (3) identified PDGF as a central contributing driver of LGLL pathogenesis in addition to IL-15 (3). This network analysis indicated that after T-cell activation, constitutive IL-15 and intermittent PDGF signaling were sufficient to reproduce known dysregulations in T-LGLL. Supporting these findings, Zhang et al. (3) found patients with T-LGLL had increased circulating levels of PDGF-BB. With the use of immunohistochemical staining, PDGF-BB protein was confirmed to be located on LGLL cells. Yang et al. (11) showed that LGLL cells have increased levels of PDGF-β receptor mRNA as compared to healthy donor cells. Treating LGLL cell lines with exogenous PDGF or serum from LGLL patients led to increased LGLL cell proliferation, which was abrogated by PI3K inhibitor (11). The authors also demonstrate that downstream targets of PDGF signaling, PI3K and Akt/ERK, are constitutively active in LGLL (11). Pharmacologic disruption of this pathway in an LGLL cell line (NKL) and primary patient samples with anti-PDGF-BB antibody led to decreases in downstream targets and increased LGLL cell apoptosis (3, 11). These findings establish PDGF as part of an autocrine loop in LGLL allowing tumor cell survival.

### Interleukin-2

Interleukin-2 (IL-2) is a 16-kDa four alpha-helix bundle cytokine in the same family as IL-15 (24). Mainly produced by activated T cells, IL-2 drives T-cell growth and differentiation *via* interaction with its heterotrimeric receptor consisting of three subunits α, β, and γ_C_ (25). IL-2R has been shown to be increased in LGLL cells (26). Yang et al. (27) investigated the link between antigen activation, IL-2, and Fas-driven death pathways in T-LGLL. Normally, IL-2 helps to initially activate T cells but then drives the cell toward apoptosis *via* activation-induced cell death (AICD). While it has been established that despite high Fas-FasL expression LGLL cells are resistant to Fas-mediated apoptosis, the connection to IL-2 signaling is not completely understood (27, 28). LGLL cells treated with exogenous IL-2 *in vitro* had restored Fas-signaling, but there was no change in c-FLIP, a protein that inhibits the formation of the death-inducing signaling complex (DISC) machinery, compared with LGLL cells untreated with IL-2. This suggests intact functioning of this pathway and, instead, a possible disruption in regulation (27). c-FLIP has been found to be overexpressed in LGLL patients, which may contribute to the cells’ resistance to Fas-induced apoptosis (27). Additionally, IL-2 signaling can activate NF-kB, Jak/STAT, and MAPK pathways, all of which can drive cell proliferation and survival (29).

### Interleukin-6

Interleukin-6 (IL-6) is a well-known pro-inflammatory, four alpha-helical, cytokine secreted by many cell types including monocytes and T cells (30). IL-6 induces Jak/STAT and Ras/Erk signaling through interactions with a unique IL-6R and membrane-bound gp130 subunits of its receptor (31). Similar to IL-15, IL-6R can be both *cis*- and *trans*-presented to the gp130 receptor subunits, which dimerize to trigger intracellular downstream signaling (30). Analyses by Teramo et al. (12) revealed that the non-leukemic cell population in patients with LGLL is more prone to producing IL-6 than the healthy counterpart. It was also shown that the high levels of IL-6 that were observed in patients with LGLL were associated with the persistent stimulation of STAT3. Inhibiting this signaling with anti-IL-6 or anti-IL-6Rα antibodies led to decreased phosphorylated STAT3 and reduced LGL survival (12). Recently, Kim et al. (32) investigated IL-6 in the plasma of T-LGLL patients (n = 9) by *STAT3* mutational status as compared to healthy donors (n = 8). They demonstrated widely upregulated cytokine profiles in the LGLL patients, specifically greatly increased IL-6 and IL-15RA, regardless of *STAT3* mutation (32).

### Miscellaneous Others

#### Interleukin-12

Early studies showed that interleukin-12 (IL-12) can act as a co-stimulatory cytokine in concert with the activation of CD3 to increase the proliferation of LGL cells *via* Jak/STAT signaling (33).

#### Interleukin-17 and Interleukin-23

Interleukin-17 (IL-17) production defines helper T cells (T_H_) and is a central pro-inflammatory driver in the immune response (34). IL-17 signaling leads to increases in granulocyte-macrophage colony-stimulating factor (GM-CSF), IL-6, monocyte chemoattractant protein-1 (MCP-1), macrophage inflammatory protein (MIP-2), and other inflammatory cytokines (34). Outlined by Zawit et al. (35), there may be potential for immunotherapeutic targeting of the IL-17/-23 signaling axis as a treatment strategy in LGLL. Interleukin-23 (IL-23) signaling through Jak/STAT receptors in T_H_17 cells can drive these cells to produce IL-17 and further perpetuate the production of pro-inflammatory cytokines (36).

#### sIL-2R, Interleukin-6, TNF-alpha, Interleukin-8, and Interleukin-10

sIL-2R, Interleukin-6, TNF-alpha, Interleukin-8, and Interleukin-10 were increased in the supernatant of LGLL primary sample cultures compared to controls (26). These cytokines can inhibit hematopoiesis, and IL-8 has been shown to lead to neutrophil extravasation. This may contribute to the neutropenia that these patients experience in addition to other autoimmune diseases (37).

#### RANTES, Interleukin-8, MIP-1 Alpha and Beta, Interleukin-10, Interleukin-18, IFNɣ, and IL1Ra

RANTES, Interleukin-8, MIP-1 alpha and beta, Interleukin-10, Interleukin-18, IFNɣ, and IL1Ra all have elevated mRNA transcripts in the PBMCs of LGL patients (38). The sera of LGLL patients demonstrated elevated levels of RANTES (Regulated upon Activation, Normal T-cell Expressed and presumably Secreted), MIP-1b, and IL-18, all of which can activate the PI3K pathway (38). Further elucidation of the mechanisms that trigger the transition from the reactive lymphoproliferation to the extreme monoclonal process and subsequent leukemogenesis revealed various phenotypic differences between the healthy and leukemic T-LGL cells. These differences include the up-modulation of various genes (IL-8, IL-18, and IFNɣ) and the presence of chemokines (MCP-1 and IP-10/CXXL10) (39). The overexpression of these chemokines and receptors (including CXCL2, hepatitis A virus cellular receptor 1, IL-18, and CCR2) in T-LGL cells are associated with viral infections. These findings support the concept that viral infections can lead to the development of T-LGL cells. Interestingly, upregulated cytokines are those typically produced by CD8+ T cells in response to viral infection, lending evidence to the idea that a virus may be triggering or perpetuating insult contributing to LGLL cell pathogenesis.

#### Epidermal Growth Factor, IP-10/CXCL10, Granulocyte Colony-Stimulating Factor

Recent serum analysis of LGLL patients by Olson et al. (40) found reduced epidermal growth factor (EGF) and increased levels of interferon gamma-induced protein 10 (IP-10) and granulocyte colony-stimulating factor (G-CSF) in LGLL serum compared to that of healthy donor controls. The authors also compared cytokine profiles between T-LGLL and NK-LGLL, which they found to be largely similar between the subtypes. They state that the reason for lowered EGF in LGLL patients is unknown but conclude increased IP-10 and G-CSF, which recruit lymphocytes and stimulate the bone marrow respectively, both fit with the clinical neutropenic context of the disease.

## Cytokine-Driven Oncogenic Pathways in Large Granular Lymphocytic Leukemia

**Figure 1:**
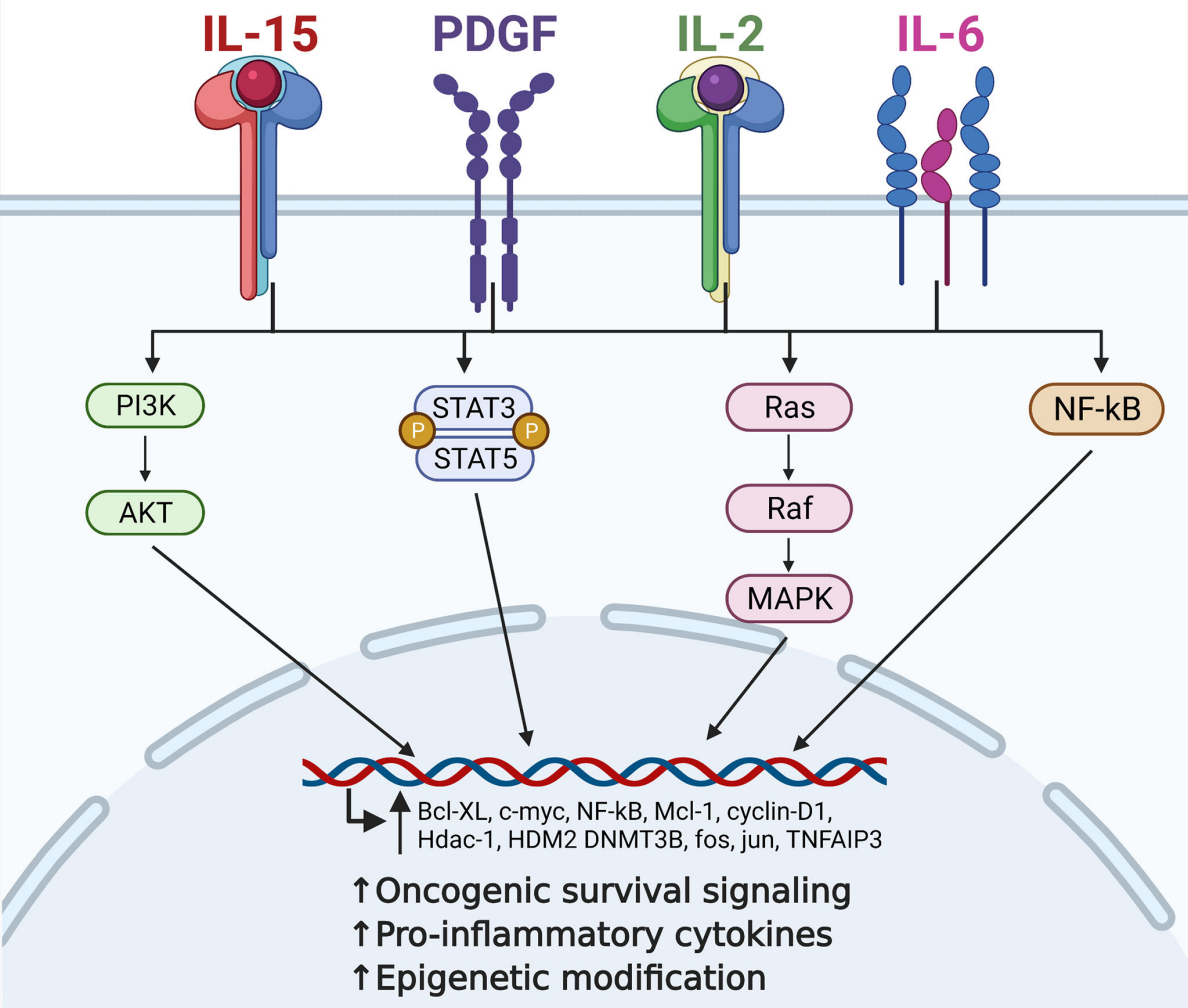
Contribution of critical cytokine signaling to large granular lymphocytic leukemia (LGLL) immunopathogenesis. Interleukin (IL)-15, platelet-derived growth factor (PDGF), IL-2, and IL-6 are all central players in the immunopathogenesis of LGLL. Dysregulation of these cytokines leads to constitutive activation of their downstream signaling pathways such as PI3K, JAK/STAT, Ras/MAPK, and NF-kB. This leads to increased transcription of oncogenic driver genes such as *c-MYC*, *cyclin D1*, and *BCL-xL*, ultimately leading to increased malignant cell proliferation and survival. Figure made with BioRender.com.

The interactions of cytokines and the downstream oncogenic signaling drivers active in LGLL are summarized below and in **Figure 1**.

### Jak/STAT

There is abundant evidence for dysregulated STAT signaling in LGLL. First described in 2001 by Epling-Burnette et al., constitutive STAT3 activation is one of the defining features of the pathogenesis of LGLL (41). Approximately 40% of T-LGLL patients have gain-of-function *STAT3* mutations and *STAT5b* variants have also been identified in LGLL subtypes (42, 43). Y640F and D661Y are the most common *STAT3* alterations, accounting for roughly 60% of cases (44). These mutations are typically found in the Src homology 2 (SH2) dimerization and activation domains of *STAT3* gene (43). The gain-of-function mutations result in stabilized dimerization, enhanced transcriptional activity, and eventually increased production of pro-survival proteins (43). In Y640F, the hydrophobic alteration to the sequence allows independent homodimerization of the protein (40). When activated, the pSTAT3 complex can then translocate to the nucleus and enhance the transcription of oncogenic driver genes such as *c-MYC*, *BCL-xL*, and *MCL1* (44).

Jerez et al. (45) linked *STAT3* mutation status to patient outcomes and clinical features showing that patients with somatic *STAT3* mutations were significantly more likely to manifest symptoms at the time of diagnosis (*p* < 0.001). These patients also typically required more treatments over the course of their disease and had a shorter “time-to-treatment-failure” than those who did not harbor *STAT3* mutations (45). The prevalence of autoimmune conditions, such as rheumatoid arthritis (RA) and autoimmune hemolytic anemia, was also higher in the *STAT3* mutated cohort. Recently, Barilà et al. (46) provided the first evidence that the presence of a *STAT3* mutation can negatively affect the survival rate of patients with LGLL (46).

To further define these clinical differences, Olson et al. (40) investigated variations in red blood cell parameters in LGLL patients grouped by *STAT3* mutation type. They found that males with D661Y *STAT3* mutations had significantly higher mean corpuscular volumes (MCVs) and lower hemoglobin levels as compared to either the Y604F group or healthy donor controls (40). This has potential implications for *STAT3* mutational status screening of LGLL patients who may present with macrocytic anemia (40). *STAT5b* mutations, N642H and Y665F, have also been found to be gain-of-function mutations in the SH2 domain and were initially discovered in a small percentage of clinically aggressive CD8+ T-LGLL (47). However, *STAT5b* mutations have subsequently also been identified in CD4+ T-LGLL patients, with incidence ranging from 15.2% to 55% reported (42, 46, 48). Clinically, these CD4+ T-LGLL patients are most often asymptomatic, without any impact on survival outcomes (46, 48). Interestingly, a recent investigation into somatic mutations of 57 NK-LGLL patients specifically showed that few (9%) had *STAT3* mutations and no *STAT5b* mutations were found (49). However, in patients negative for *STAT3* mutations, the authors observed mutations in many other genes related to cancer pathogenesis, including those related to Ras/MAPK and PI3K/Akt signaling, as well as *TET2*, which plays a role in epigenetic modification (49). *STAT3* mutations have also been identified in ~43% of patients with Felty syndrome (FS; a rare disease that shares many clinical similarities with LGLL), as well as significant increases in ten cytokines common to both LGLL and FS (50). IL-15Ra, IL-6, MIP-1a, CXCL10, and CSF-1, as well as oncostatin-M, TNFRSF9, PD-L1, CDCP1, and HGF, were those notably upregulated in both FS and LGLL, further emphasizing the link between cytokine and STAT3 dysregulation and disease pathogenesis (50). These differences in mutational landscape delineated by the immunophenotype of the malignant cells are interesting to consider and may have future applications with regard to disease screening or treatment strategy in the age of precision medicine.

Regardless of mutational status, all LGLL patients have constitutively upregulated STAT3 activity, in large part due to pro-inflammatory cytokine drivers. As previously discussed, IL-15 and IL-6 are both increased in LGLL patients and are known activators of Jak/STAT signaling. There is evidence for IL-15 as a central pathogenic driver in LGLL initiation and progression through Jak/STAT signaling (3, 10). Physiologically, it is important to note that while short-term exposure to IL-15 increases proliferation, survival, and cytotoxic activities of LGL cells, long-term chronic activation of STAT by IL-15 has been shown to be leukemogenic (10). As described in Fehninger et al. (51), mice that were engineered to overexpress IL-15 develop spontaneous fatal LGLL. However, it is interesting to note that *STAT3* mutations alone are not sufficient to induce LGLL in a mouse model, suggesting that cytokine signaling and other pathway dysregulations are critical for oncogenesis (52).

### Ras-Raf-1-MEK1-ERK/MAPK

IL-2, IL-6, IL-15, and PDGF can all activate the Ras-Raf-1-MEK1-ERK/MAPK signaling pathway. Ras and ERK have been found to be constitutively active in NK-LGLL. The aggressive LGLL cell line, PLT-2, has a G12A *KRAS* mutation (53, 54). Mizutani et al. (54) postulated that it is the *KRAS* mutation that allows the PLT-2 cell line to grow independently from any exogenous IL-2 stimulation, unlike MOTN-1, a chronic T-LGLL line, which requires IL-2 and IL-15 cytokine stimulation for survival. Inhibiting Ras in LGLL cells with a farnesyltransferase inhibitor, FTI2153, caused ERK inhibition and induced apoptosis *via* Fas signaling and independently of Fas (53). Inhibition of MEK1 also reduced the survival of NK-LGLL cells (53). All of this suggests that dysregulation of this pathway may have both pro-growth and anti-apoptotic influences on LGLL cell pathogenesis. The exact mechanisms by which MEK/ERK signaling are driving LGLL cell survival are not yet fully defined. However, it has been established that activated MAPK is capable of regulating anti-apoptotic proteins. For example, Bcl-2, BAD, and p-ERK can phosphorylate proto-oncogenic transcription factors in the nucleus such as Fos and Jun (53, 55). The Ras cascade also has the ability to crosstalk with PI3K/Akt signaling, further affecting downstream signaling in LGLL pathogenesis (56).

### PI3K/Akt

Activated by Ras signaling, and PDGF, as well as IL-18, RANTES, and MIP-1, the PI3K/Akt signaling pathway is a major driver of pro-survival signaling in LGLL (3, 38). Compared to healthy donors, T-LGLL cells have increased PI3K/Akt activity, as indicated by higher levels of p-Akt, which contributes to downstream resistance to apoptosis (56). p-Akt can activate mTOR, a major driver of cell growth and proliferation (57). Schade et al. (58) show that Src family kinases can lead to constitutive activation of the PI3K pathway in LGLL, eventually leading to anti-apoptotic signaling *via* disruption of DISC formation. This effect was abrogated using a PI3K inhibitor, LY294002, which restored apoptosis and showed a reduction in ERK expression, reinforcing the concept of crosstalk between these two pathways (58).

Akt can also interfere with the regulation of transcription factor NF-kB by blocking its inhibition. This leads to increased NF-kB activity and enhanced transcription of oncogenic genes (59). Administration of the PI3K inhibitor LY294002 also resulted in significantly decreased NF-kB activity in T-LGLL cells as well as cell apoptosis, and one of two LGLL patients treated on a phase I study of the dual PI3K δ/γ inhibitor duvelisib had a prolonged partial response (3, 60).

### NF-kB

NF-kB is a transcription factor that regulates the survival of immune cells and can be activated by IL-15, Ras, and Akt/PI3K. It can translocate to the nucleus, activating the transcription of pro-survival and anti-apoptotic genes, such as *cyclin D1*, *c-MYC*, *BCL-2*, and *MCL-1*, and can induce the production of IL-2 (41, 61–63). Zhang et al. (3) compared nuclear extracts of T-LGLL cells to nuclear extracts of healthy donor PBMCs and found that c-Rel, an NF-kB family protein, is increased and constitutively active in T-LGLL. When NF-kB was inhibited, the T-LGLL cells had significantly induced apoptosis that was not observed in normal healthy donor PBMCs (*p* < 0.009) (3). Interestingly, the authors also showed that the Mcl-1-driven pathogenic effect of NF-kB in T-LGLL can occur independently of STAT3 signaling, adding another facet of possible signal compensation to this complicated disease picture. Recently, Olson et al. (64) identified missense mutations in *TNFAIP3*, a negative regulator and target of NF-kB, in 8% of a cohort of 39 LGLL patients (64, 65). *TNFAIP3* expression has been previously shown to be upregulated in LGLL samples, further emphasizing the importance of NF-kB signaling in LGLL pathogenesis (65, 66). Yang et al. (67) established a link between the cytokine TRAIL (Tumor Necrosis Factor-Related Apoptosis-Inducing Ligand) and NF-kB in LGLL by demonstrating increased TRAIL mRNA and protein in LGLL cells as well as increased soluble TRAIL in sera from LGLL patients compared to healthy controls (67). TRAIL can bind death receptors to induce apoptosis in tumor cells as well as activate the NF-kB pathway. The LGLL cells express the TRAIL receptor DcR2, and activation of this receptor by TRAIL leads to increases in NF-kB signaling. Through this mechanism, NF-kB’s pro-survival and anti-apoptotic activities are further driven by cytokine signaling in LGLL.

## Interaction of Oncogenic Drivers and Cytokine Signaling Pathways

The frequent co-existence of dysregulated cytokine signaling and oncogenic mutations has been described in LGLL. The *TNFAIP3* missense mutations in NF-kB signaling observed by Johansson et al. (66) were significantly associated with *STAT3* mutations in LGLL patient samples, a combination also seen in other lymphomas (66). Coppe et al. (68) identified *CD40LG* as a mutated receptor in LGLL patient samples. CD40LG is involved in STAT3 signaling, as well as MAPK-Ras-Erk, and the IL-15 pathways. Interestingly, *CD40LG* mutations were also seen as functionally related to *TNFAIP3* in the analysis, meaning that there is also a potential link to NF-kB signaling dysfunction. In addition to *CD40LG* lesions, the authors identified activating mutations in *FLT3* receptor tyrosine kinase, which has implications for Ras, Jak/STAT, and PI3K/Akt signaling (68).

It has been established that increased IL-15 can affect the expression of Bcl-2 family genes. However, Hodge et al. (19) further elucidated a mechanism by which IL-15 may be driving anti-apoptotic signaling in LGLL pathogenesis. The authors demonstrate that IL-15 causes upregulation of HDM2, a p53-E3 ligase, which can drive proteasomal degradation of Bid, a protein that is essential for cell apoptosis (19). Through this mechanism, IL-15 can reduce Bid in T-LGLL and NK-LGLL samples. Inhibiting IL-15 or the proteasome degradation pathway in these samples restored Bid levels and showed increased cell death (19).

Previous work from Mishra et al. (10) demonstrates how chronic IL-15 exposure can initiate LGLL through NF-kB signaling and Myc induction in tumor cells. In normal wild-type mouse LGL cells treated with IL-15, this cytokine induces Myc expression *via* the NF-kB pathway. Myc was then shown to mediate increases in aurora kinases A and B. Elevation of *AURKA*, *AURKB*, and *MYC* was confirmed in primary LGLL patient samples, and Myc knockdown in mouse LGL cells showed reduced *AurkA* and *AurkB*. The increased aurora kinases led to centrosome aberrations and result in chromosomal aneuploidy, which is a consistent finding in patient LGLL cells. This chromosomal instability helps drive leukemic oncogenesis. Concurrent to aurora kinase upregulation, the IL-15-driven induction of Myc, NF-kB, and Hdac-1 results in the reduction of *miR-29b* when these repressor proteins bind to its promotor. Indeed, *miR-29b* levels were demonstrated to be significantly decreased in LGLL patients (*p* < 0.0009) as well as healthy donor LGL cells exposed to IL-15 (*p* < 0.003). *Mir-29b*, in turn, typically negatively regulates Dnmt3b, a DNA methyltransferase, with the expression of *DNMT3B* found to be elevated in primary LGLL patient cells. Thus, the increased Dnmt3b in LGLL results in DNA hypermethylation, leading to further chromosomal instability as well as possible silencing of tumor suppressor genes (10). Mishra et al. (10) further demonstrated increased global DNA methylation in primary samples from LGLL patients, as well as healthy LGL cells treated with IL-15 *in vitro* to support this. Through these mechanisms, it is clear that IL-15 has a critical role in the pathogenesis of LGLL.

In addition to *miR-29b*, another miRNA has recently been implicated in the pathology of LGLL. Mariotti et al. (69) identified reduced expression of *miR-146b* in CD8+ T-LGLL due to *miR-146b* promotor hypermethylation. This observed repression of *miR-146b* expression was dependent on STAT3 activation, likely *via* the action of DNMT1, and could be experimentally reversed in CD8+ T-LGLL cells by inhibiting STAT3 (69). Interestingly, the authors also demonstrate how miR-146b may contribute to the development of neutropenia in LGLL *via* interaction with Fas-ligand signaling. Absolute levels of neutrophils in LGLL patients correlated with miR-146b levels and are inversely correlated with the amount of soluble Fas-ligand (FasL) (69). The authors posit that miR-146b-target protein HuR is increased in CD8+ T-LGLL, which serves to stabilize the translation of FasL, ultimately leading to increased levels of FasL in this disease and a mechanism for the resultant neutropenia. In this way, cytokine drivers of STAT3 activation can further alter miRNA levels to drive LGLL pathogenesis.

The loss of suppressor of cytokine signaling-3 (SOCS3) may also be contributing to the pathogenic potential of IL-6 signaling in LGLL. SOCS3 is typically induced by IL-6 *via* p-STAT3. However, despite the upregulated levels of IL-6 and STAT3 observed in LGLL, Teramo et al. (12) found a decreased amount of SOCS3 mRNA and protein in LGLL patient samples compared to healthy donors. Typically, SOCS3 is responsible for negatively regulating Jak/STAT signaling. The authors demonstrated that SOCS3 does not respond appropriately to p-STAT3/IL-6 messaging in the LGLL cells, which may further drive dysregulated STAT signaling. However, after treating the LGLL cells with decitabine, a demethylating compound, appropriate IL-6-driven increases of SOCS3 mRNA and protein were observed (12). This treatment also correlated with decreased p-STAT3, decreased Mcl-1, and increased LGLL apoptosis. Decitabine’s effective mechanism of action, demethylation, lends support to the conclusion that epigenetic changes may be silencing normal SOCS3 responses in LGLL. However, abnormal methylation changes to the SOCS3 promoter were not seen, leading the authors to conclude that epigenetic modification occurs elsewhere (12). In this way, IL-6 and loss of the SOCS3 regulator work together to further drive Jak/STAT signaling and LGLL pathogenesis.

Olson et al. (64) recently investigated epigenetic changes in NK-LGLL patient samples. Methylation of *TET2* promoter sequences as well as hypermethylation of negative regulators of *STAT3*, *PTPRD*, and *PTPRN* was observed. TET2 typically contributes to DNA demethylation. This study also identified loss-of-function mutations in this gene in 28% of their observed NK-LGLL patients (n = 58). These patients had significantly increased global methylation compared to healthy controls (64). Thus, in addition to driving increased STAT activation, epigenetic modification may also be facilitating further enhanced methylation of the genome in LGLL. Another study analyzed the *TET2* mutational hierarchy in NK-CLPD by performing whole-exome sequencing of different hematopoietic cells (70). It revealed that the *TET2* alteration was shared by NK-LGLL and cells of the myeloid compartments. This study concluded that the multi-hit model could explain the emergence of *TET2* mutations during the early stages of hematopoietic progenitors (70). *TET2* mutations were also associated with the CD16^low^ phenotype in NK-LGLL (70).

Kim et al. (32) recently demonstrated how cytokine and epigenetic changes in LGLL can be regulated by STAT3 activity. This study demonstrated that IL-15 mRNA expression levels are significantly higher in STAT3 mutated LGLL. Additionally, T-LGLL patient samples with *STAT3* mutations had high STAT3 levels and increased pSTAT3 compared to healthy controls. Additionally, increased DNMT1, DNMT3, EZH2, and MYC protein were seen in T-LGLL compared to controls. DNMT1, DNMT3, and EZH2 are methyltransferase enzymes that can affect epigenetic modifications. These findings were recapitulated in KAI3 NK cells with *STAT3*^Y640F^ or *STAT3*^G618R^ mutations, and increased p65, a subunit of NF-kB, highlighting the crosstalk potential between these signaling pathways. Treatment of healthy donor CD8+ T cells with IL-6, IL-15, and MCP-1 cytokines led to enhanced phosphorylation of STAT3 and increased DNMT1, DNMT3B, and EZH2 protein. This further defines a mechanistic link between cytokine signaling and regulators of epigenetic modification. This study also observed direct binding of mutated STAT3 to DNMT1 and EZH2 protein, further defining the mechanism of action of this pathway. Treating *STAT3*-mutated LGLL cells with hypomethylating agent 5-azacytidine led to reduced cell viability, STAT3 phosphorylation, and DNMT1 (32, 71). Through these results, the authors define how cytokine signaling and STAT3 mutations in LGLL can directly drive epigenetic changes in this disease, clarifying new targets for further investigation and potential therapeutic intervention.

## Mechanisms of Cytokine Dysregulation in Large Granular Lymphocytic Leukemia

While it is established that cytokine signaling is involved in LGLL initiation and maintenance, how the cytokines involved become upregulated is not well characterized. The working theory for the initiation of LGLL involves an antigenic insult that triggers an inflammatory state and immune cell reactivity that gets inappropriately perpetuated through a variety of signaling and genetic mechanisms (9). It is likely that to some degree, the hyperactivation of signaling pathways such as Jak/STAT, Ras-Raf-Mek-Erk, PI3K, and NF-kB further drives cytokine production, release, and response in a feed-forward loop. However, exact details have not been thoroughly elucidated. IL-6 signaling, for example, induces STAT3, which has the ability to promote *IL-6* gene expression in an autocrine feed-forward loop, but this has yet to be demonstrated conclusively in LGLL (72, 73).

In addition to signaling deficiencies, mutations and epigenetic changes may also contribute to cytokine dysregulation in LGLL. Previous work has shown some evidence for hypermethylation of the IL-15 promotor in LGLL patient samples compared to healthy donor cells (71). Mishra et al. (74) have previously shown increased IL-15 promoter methylation in cutaneous T-cell lymphoma (CTCL), another T-cell malignancy largely driven by IL-15 pathogenesis. In the case of CTCL, the hypermethylation prevents repressor protein binding and results in aberrantly increased IL-15 expression. In LGLL cell line samples, treatment with 5-azacytidine (a hypomethylating agent) resulted in decreased IL-15 gene expression and decreased cell viability, lending evidence to epigenetic changes contributing to IL-15 overexpression in LGLL (71).

*PDGF* and *PDGFR* genetic and epigenetic alterations have been described previously in other hematologic malignancies but have yet to be characterized in LGLL (75, 76). Changes to PDGF receptor proteins may allow for ligand-independent activation and escape from inhibitory mechanisms or degradation pathways. Possible changes to this pathway need further investigation in the setting of LGLL, given the central role of PDGF signaling in disease pathogenesis (3).

There is clear evidence that overproduction of cytokines can lead to the development of LGLL and various types of cytopenia in patients with LGLL. The challenge in treating patients with a heterogeneous disease like LGLL is to identify patients who may benefit most from blocking the activity of cytokines. The use of targeted approaches for the neutralization of oncogenic or immunosuppressive cytokines could provide new opportunities to develop effective therapeutic strategies for LGLL patients.

## Cytokine-Driven Animal Models of Large Granular Lymphocytic Leukemia

The use of animal models of LGLL has greatly enriched our understanding of the pathogenesis of LGLL and provided the opportunity to test novel therapeutics in the disease context. Fehniger et al. (51) developed a transgenic mouse that overexpressed IL-15 by removing posttranscriptional checkpoint inhibitors, allowing for more efficient translation and secretion. These mice developed fatal lymphocytic leukemia between 12 and 30 weeks of age with an NK-T signature of CD3+TCRB+DX5+ markers (51). Phenotypically, the mice developed alopecia, hepatosplenomegaly, weight loss, and extreme clonal lymphocyte expansion in blood, spleen, and bone marrow. The authors described a “blast morphology” of these lymphocytes, which infiltrated many organ systems (77). This model best recapitulates the aggressive T and NK variants of LGLL. This chronic upregulation of IL-15 can induce oncogenic signaling pathways to drive the development of LGLL (10).

Klein et al. (78) described a mouse model that expresses the human STAT5B^N642H^ mutation, which goes on to develop CD8+ T-cell leukemia (78, 79). This stands in contrast to a study by Dutta et al. (52), which demonstrated that activating STAT3 mutations in mice was not sufficient to induce LGLL. The STAT5B^N642H^ lesion is a gain-of-function mutation in the SH2 domain. Similar to the IL-15 transgenic mice, both models have leukemic immunophenotypes positive for CD122, NKp46, and DX5, mirroring CD3+NK1.1+ T-LGL cells (77, 78). The authors also showed that these STAT5B^N642H^ mutation mice could be successfully treated with ruxolitinib, a JAK inhibitor, further emphasizing the central role of dysregulated STAT signaling in LGLL pathogenesis (78).

## Therapeutic Blocking of Cytokine Signaling in Large Granular Lymphocytic Leukemia Treatment

**Figure 2:**
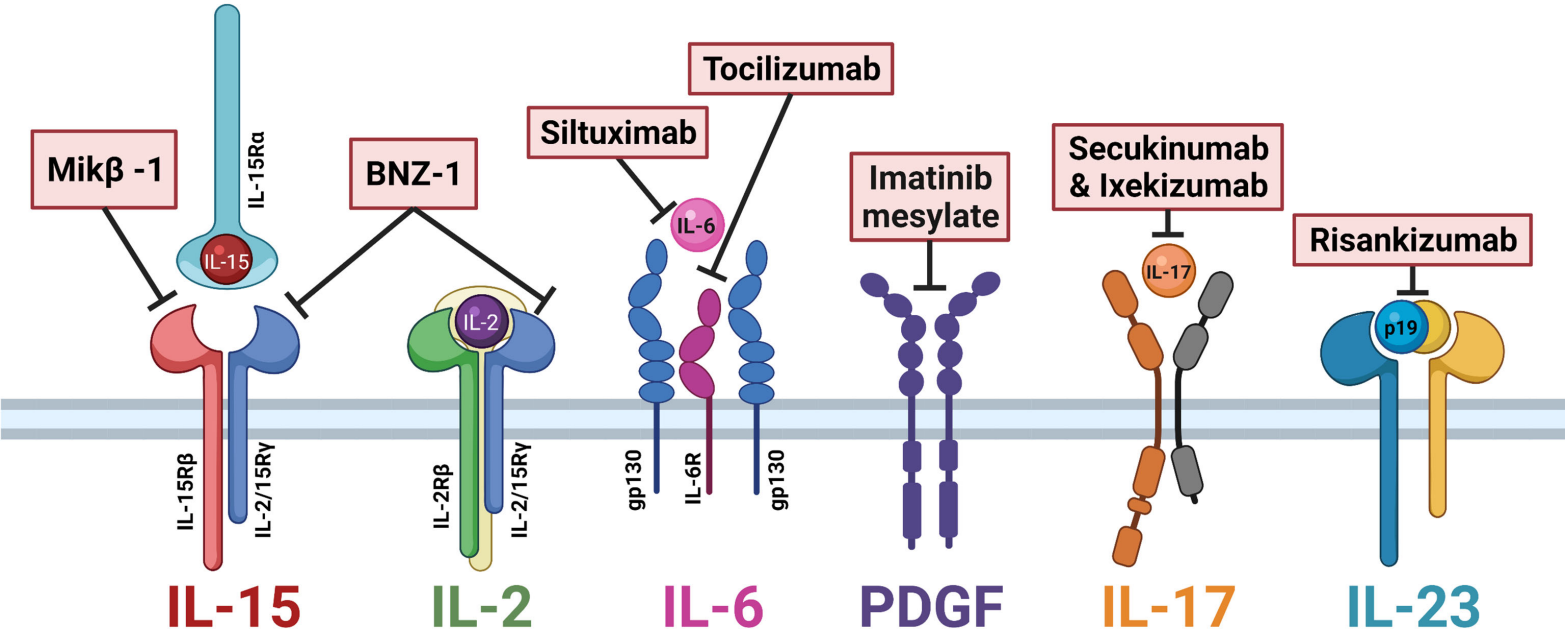
Cytokine-directed therapies of interest in large granular lymphocytic leukemia (LGLL). Mik-β1, a CD122 monoclonal antibody, prevents the *trans*-presentation of interleukin (IL)-15. BNZ-1 binds the common gamma chain CD132, blocking IL-15 and IL-2 signaling. Siltuximab and tocilizumab block IL-6 signaling. Imatinib mesylate is a receptor tyrosine kinase inhibitor that prevents platelet-derived growth factor (PDGF) signaling. Secukinumab and ixekizumab block IL-17 signaling. Risankizumab binds the p19 subunit of IL-23 to block signaling. Figure made with BioRender.com.

Currently, LGLL is not a curable disease, and the mainstay of treatment remains general immunosuppressive therapy. Frontline agents include methotrexate, cyclophosphamide, and cyclosporine, whose efficacy is typically limited to partial remissions (60). However, with new insights into LGLL pathogenesis, researchers have brought novel targets of clinical interest into pharmaceutical development. Of particular interest is those targeting cytokine signaling, which are outlined in **Table 1** and summarized in **Figure 2**.

**Table 1:** Therapies targeting cytokine signaling in Large Granular Lymphocytic Leukemia.

Therapeutic agent	Mechanism/findings	Reference
**Agents tested against LGLL *in vivo***		
**Hu-Mikβ1**	Anti-CD122 (shared IL-2 and IL-15 receptor β-chain) monoclonal antibody. Blocks *trans* presentation of IL-15 to T cells. In a phase I study in LGLL, the drug was safe but showed no clinical efficacy.	(80)
**BNZ-1**	Multi-cytokine inhibitor that prevents IL-2, IL-9, and IL-15 from interacting with the gamma receptor subunit CD132. Wang et al. (81) demonstrated that treating T-LGLL cell lines and primary patient samples with BNZ-1 led to reduced tumor cell viability, decreased downstream signaling, and increased apoptosis. Additionally, Brammer et al. (83) showed apoptosis of LGLL cells in patients treated with BNZ-1 within 24 h of treatment. A phase I/II clinical trial (NCT03239392) showed a 90% decline in T and NK cells by day 15 of treatment (82).	(81–83)
**5-azacytidine**	Hypomethylating agent: treatment of the LGLL cell line MOTN-1 cells with 5-azacytidine resulted in decreased IL-15 expression; implicating IL-15 promoter hypermethylation as a key driver of IL-15 induced LGLL. Decreasing IL-15 production by demethylating the promoter is being explored in a phase I clinical trial (NCT05141682) evaluating an oral 5-azacytidine formulation (CC-486) in patients with LGLL.	(71)
**Agents tested against LGLL *in vitro***		
**Siltuximab and tocilizumab**	Anti-IL-6 and anti-IL-6R, monoclonal antibodies currently approved for treatment of rheumatoid arthritis by inhibiting JAK pathway signaling. *In vitro* anti-IL-6 antibody treatment of LGLL patients’ PBMCs led to malignant cell apoptosis (12).	(12, 84)
**Agents of interest in LGLL**		
**Imatinib mesylate (STI-571)**	A receptor tyrosine kinase inhibitor that can target PDGF receptors.	(85)
**Secukinumab and ixekizumab**	Anti-IL-17 monoclonal antibodies that prevent IL-17 receptor binding and downstream JAK/STAT and NFkB signaling. Currently, FDA-approved for ankylosing spondylitis and psoriatic arthritis treatment.	(86)
**Risankizumab**	Anti-IL-23 humanized monoclonal antibody binds the p19 subunit of IL-23 to block signaling. Currently, FDA-approved for plaque psoriasis treatment.	(87)

LGLL, large granular lymphocytic leukemia; IL, interleukin; PBMCs, peripheral blood mononuclear cells; CML, chronic myelogenous leukemia; FDA, Food and Drug Administration.

Cytokine-directed therapeutic agents that have been tested against LGLL *in vivo* include Hu-Mikβ1, BNZ-1, and 5-azacytidine. Hu-Mikβ1 is a monoclonal antibody against CD122, the shared β-chain receptor for IL-2 and IL-15 (80, 88). In a phase I clinical trial of Hu-Mikβ1 in LGLL patients, Waldmann et al. (80) observed that Hu-Mikβ1 blocked the *trans* presentation of IL-15 to T cells but did not affect *cis* signaling. The authors demonstrated the safe use of Hu-Mikβ1 but did not find any clinical efficacy in LGLL patients (80).

BNZ-1 is a peptide that binds the common gamma-chain receptor CD132 and prevents IL-2, IL-9, and IL-15 signaling (81). Wang et al. (81) treated LGLL cell lines and primary patient samples with BNZ-1 and showed that in both cases tumor cell viability decreased and apoptosis increased. Additionally, BNZ-1 blockage of IL-2 and IL-15 signaling led to reductions in downstream mediators of these cytokine pathways such as p-STAT, p-Akt, and p-ERK targets (81). *In vivo*, inhibition of IL-15 using BNZ-1, as part of a phase-I/II clinical trial (NCT03239393), resulted in apoptosis of LGLL cells in nearly all patients within 24 h of administration and clinical responses in 20% of patients, clearly demonstrating the crucial role of this cytokine to LGLL pathogenesis and potential clinical value of this therapy (82, 83).

5-Azacytidine is a hypomethylating agent that decreased IL-15 expression and reduced cell viability in the MOTN-1 LGLL cell line (71). This evidence further implicates hypermethylation as a central driver of IL-15 and LGLL pathogenesis. The oral formulation of this potential treatment is currently being investigated in phase I/II clinical trial (NCT05141682) in LGLL patients (71).

Potential therapeutics that have yet to be tested in humans but have shown efficacy *in vitro* are siltuximab and tocilizumab. Siltuximab and tocilizumab are monoclonal antibodies against IL-6 and IL-6R, respectively. Currently approved for the treatment of RA, they inhibit JAK pathway signaling. Treating LGLL patients’ PBMCs with anti-IL-6 antibodies led to malignant cell apoptosis (12). The co-occurrence of some LGLL patients with RA or RA-like symptoms may especially make this line of treatment inquiry worthwhile for further investigation.

Agents of interest in LGLL that have yet to be tested in this disease but align with known LGLL pathogenic mechanisms are imatinib mesylate (STI-571), secukinumab and ixekizumab, and risankizumab. Imatinib mesylate (STI-571) is a receptor tyrosine kinase inhibitor that can target the PDGF receptor to inhibit signaling (85). While typically used in chronic myelogenous leukemia (CML), the known pathogenic role of PDGF signaling in LGLL warrants further investigation into the usefulness of targeting this cytokine pathway (3). Secukinumab and ixekizumab are both monoclonal antibodies that prevent IL-17 receptor binding and limit downstream JAK/STAT and NFkB signaling (86). Given the role of IL-17 as a pro-inflammatory chemoattractant implicated in the pathology of various autoimmune conditions (which afflict a subset of LGLL patients), blocking IL-17 signaling is a strategy worth exploring in the setting of LGLL. Similarly, IL-23 can signal through Jak/STAT receptors in T_H_17 cells to drive these cells to produce IL-17, thereby further perpetuating the inflammatory milieu (36). Risankizumab is a monoclonal antibody against IL-23 that binds the p19 subunit of IL-23 to block signaling (87). The IL-17/IL-23 signaling axis constitutes an intriguing target of therapeutic intervention for LGLL based on its known role in driving inflammation and autoimmune conditions. In summary, there are several novel cytokine-related treatment strategies worth further investigation in LGLL.

## Conclusion

Aberrant cytokine expression and signaling are important components of LGLL pathogenesis. It is not yet clear how effective interventions that target inflammation will be in preventing the onset and/or progression of LGLL. Understanding these cytokine signaling pathways and their various components will help develop novel therapeutic agents and treatment strategies. The re-establishment of cytokine homeostasis in LGLL could benefit patients who suffer from this disease, especially those refractory to current therapeutic options.

## Author Contributions

CI, JB, and AM planned and conceptualized the review. CI wrote the initial draft. CI, AM, JB, AB, NC, and PP contributed to writing, review, and revision. All authors listed have made a substantial, direct, and intellectual contribution to the work and approved it for publication.

## Funding

Work supported by American Society of Hematology grant to AM and National Cancer Institute grant to AM.

## Conflict of Interest

JB, AM, and PP have received funding from pharmaceutical companies for research and clinical trials.

The remaining authors declare that the research was conducted in the absence of any commercial or financial relationships that could be construed as a potential conflict of interest.

## Publisher’s Note

All claims expressed in this article are solely those of the authors and do not necessarily represent those of their affiliated organizations, or those of the publisher, the editors and the reviewers. Any product that may be evaluated in this article, or claim that may be made by its manufacturer, is not guaranteed or endorsed by the publisher.
